# Professional ethics cultivation as a driver of faculty development: an analysis of its four underlying logics

**DOI:** 10.3389/fpsyg.2026.1840284

**Published:** 2026-09-11

**Authors:** Jiang-bo Bai

**Affiliations:** 1 School of Marxism, Guizhou Medical University, Guiyang, China; 2 School of Politics and Public Administration, Shandong University, Jinan, China

**Keywords:** conceptual analytical framework, faculty development, four logics, internal relationship, professional ethics cultivation, university faculty

## Abstract

**Introduction:**

Professional ethics cultivation serves as a vital approach to building a high-quality teaching workforce, exerting a foundational and endogenously driving effect on the development of university faculty.

**Methods:**

Grounded in the perspective of individual teachers’ self-moral cultivation, this study employs theoretical analysis to construct a four-dimensional logical framework through which professional ethics cultivation empowers faculty development.

**Results:**

The analysis reveals four core endogenous driving forces: professional ethics cultivation functions as (1) a process factor for faculty intensive development, (2) an embodiment of the internalization of faculty professional attributes, (3) an inherent requirement for faculty professional growth, and (4) a resource endowment for faculty career advancement. Against the global backdrop of high-quality higher-education construction and standardized governance of faculty professional ethics, existing studies predominantly explore professional ethics cultivation from the perspectives of institutional management and training effectiveness, yet overlook the endogenous mechanism through which such cultivation fosters faculty comprehensive growth.

**Discussion and conclusion:**

The proposed four-dimensional framework integrates individual moral growth, professional-identity formation, endogenous career motivation, and long-term career competitiveness into a unified analytical system. This framework clarifies the internal laws of university faculty development, advances the high-quality construction of teaching teams, and provides a novel conceptual analytical tool for subsequent empirical research on faculty professional ethics cultivation.

## 1 Introduction

High-quality teachers must possess sound moral cultivation together with sophisticated teaching skills. Moral cultivation concerns individuals’ inner character and disposition. The professional ethics cultivation of university teachers refers to “the activity of transforming university teachers’ professional-ethics requirements into personal beliefs and putting such beliefs into practice; simply put, it is a form of self-directed moral education.” Evidently, university teachers’ professional ethics cultivation is mainly shaped by the particularity of the teaching profession and external expectations associated with teachers’ social roles. For individual university teachers, strengthening professional ethics cultivation realizes the elevation of their inner essence. For universities, it constitutes the principal guarantee for the quality of teaching and learning. For society and the nation, it embodies social harmony, educational prosperity, and the intergenerational transmission of knowledge and culture. Therefore, an in-depth understanding of the intrinsic logic between professional ethics cultivation and university faculty development enables university administrators to follow laws of faculty development, leverage the functional mechanism of professional ethics cultivation, enhance teachers’ comprehensive qualities, and build a high-quality teaching workforce.

## 2 Literature review and research questions

### 2.1 Research on professional ethics cultivation and faculty development

University teachers’ professional ethics cultivation stands as a core category in educational ethics. It also acts as a critical variable determining higher-education quality, standards of scientific-research innovation, and the effectiveness of talent cultivation. In the new-era context, faculty professional ethics has evolved from traditional occupational norms into a complex multi-dimensional structure covering psychological components, relational domains, and professional identification. The level of ethics cultivation directly constrains teachers’ pathways toward professionalization and the health of educational ecosystems (Liu, 2024a). Recent studies confirm that constructing a scientific professional-ethics assessment framework and strengthening teachers’ ethical awareness play a foundational role in improving the overall governance performance of universities (Liu et al., 2026).

#### 2.1.1 Re-conceptualizing professional ethics cultivation and multi-dimensional assessment systems

Traditional perspectives often reduce faculty professional ethics to behavioural codes or moral exhortation. Recent scholarship, however, identifies it as a core indicator of teachers’ professional maturity, encompassing comprehensive advancement from professional-identity recognition to ethical decision-making capacity (Liu, 2024a). Liu et al. (2026) developed a two-dimensional measurement framework containing four psychological components and four relational domains. Using mixed qualitative-and-quantitative methods, they specified constitutive elements of university teachers’ professional ethics and laid an empirical foundation for quantitative assessment. Moving beyond prior research focused merely on external behavioural constraints, their framework elaborates logical relationships between teachers and students, teachers and institutional collectives, teachers and teaching practice, as well as teachers and academic research (Liu et al., 2026).

Furthermore, ethical competence is regarded as a fundamental component of teacher professionalism, whose formation demands systematic cultivation mechanisms (Kuchai et al., 2022). From a sociology-of-knowledge perspective, faculty professional ethics is socially constructed and context-dependent; its meanings dynamically evolve alongside historical shifts and contemporary social demands (Cheng and Qi, 2022). For instance, in the digital-era context, teachers face new ethical challenges such as data privacy risks, algorithmic bias, and virtual-space interaction. Consequently, professional ethics cultivation must integrate digital literacy and ethical boundary-setting (Liu and Liu, 2024).

#### 2.1.2 Substantive impacts of professional ethics cultivation on talent cultivation and academic ecosystems

University teachers’ professional-ethics level positively predicts students’ academic outcomes. Empirical work by Sajid et al. (2024) demonstrates that university teachers’ professional ethics exerts critical influence on establishing interpersonal trust, safeguarding academic integrity, and stimulating students’ learning motivation. In private higher-education settings, faculty professional ethics and student mental-health mutually reinforce each other: sound professional ethics can mitigate students’ psychological stress and support their holistic development (Ke, 2024).

For scientific research and academic ecosystems, professional ethics cultivation forms the fundamental line of defence against academic misconduct and preserves research integrity. With market-economy development and increasingly complex social-interest structures, some university teachers exhibit declining professional ethics (Kong and Kong, 2019). Enhancing professional ethics cultivation matters not only for individual faculty careers but also for national talent-cultivation quality and social contribution (Wang, 2024). Studies reveal that differentiated leadership styles moderate teachers’ moral cognition and further shape their ethical conduct, indicating complex interaction between organizational contexts and professional ethics cultivation (Zhu and Guo, 2021).

#### 2.1.3 Practical dilemmas facing university teachers’ professional ethics cultivation in the new era

Despite institutional progress in faculty-ethics construction, multiple practical dilemmas persist. First, the cultivation of professional ethics among early-career teachers remains weak, manifested in diluted professional sentiment and moral awareness, inadequate service commitment (Zhang and Chen, 2019), vague professional ideals and convictions (Xue-nan and Li, 2020). Second, the impact of pluralistic cultural values drives some teachers toward ethical relativism and blurred moral boundaries (Liu and Jiang, 2024). Additionally, moral challenges persist within continuing education: financial constraints, heavy workloads, and geographical barriers prevent teachers in remote regions from accessing professional training and updating their ethical capacities (Ann Eloisa, 2025).

#### 2.1.4 Optimization pathways: systematic construction from institutional constraints to inner consciousness

Multiple-dimensional strategies are required to enhance university teachers’ professional ethics cultivation. First, improve institutional and legal frameworks, establish regular ethics evaluation and supervision mechanisms, and render professional-ethics norms operable (Wang et al., 2023). Second, foster teachers’ self-cultivation and inner identification. Narrative inquiry and similar approaches can guide teachers to reflect on professional experiences and support the transition from pre-service education toward lifelong professional development (Harðarson and Magos, 2021). Third, build collaborative cultivation mechanisms that integrate craftsmanship (Bu, 2020), professional motivation, and professional identity so as to strengthen teachers’ sense of occupational belonging (Huang and Espeso, 2024).

Specialized professional-ethics training programmes should target early-career teachers, incorporating curriculum-based ideological and political education to reinforce professional ethics in teaching practice (Ma et al., 2025). Meanwhile, technological advantages of the intelligent era can be leveraged to innovate ethics-education modes (Liu, 2024b). Finally, supportive organizational cultures should be cultivated, teachers’ subjective status in ethics education should be acknowledged, institutional constraints should be alleviated, and teachers’ moral potential should be fully unlocked (Saada, 2024).

### 2.2 Research review and research questions

To sum up, professional ethics cultivation constitutes the soul and cornerstone of faculty development for university teachers. Developing sound assessment systems, addressing practical dilemmas, and implementing systematic optimization strategies can effectively enhance professional ethics cultivation and advance high-quality higher-education development.

Existing literature presents the following characteristics. First, most studies adopt external administrative perspectives and rarely analyse professional ethics cultivation through the lens of teachers’ endogenous individual growth. Second, few studies address the comprehensive development of university teachers and lack integrative theoretical frameworks covering teachers’ full career cycles. Third, scholars seldom differentiate the differentiated functions of professional ethics cultivation across teachers’ developmental stages; systematic multi-dimensional logical generalizations remain scarce.

Against this backdrop, this paper puts forward three research questions:

1. What intrinsic mechanism connects university teachers’ professional ethics cultivation to their comprehensive career development?
2. What underlying logic enables professional ethics cultivation to support faculty intensive development and sustainable growth?
3. What theoretical insights and practical implications can this logical framework deliver for university faculty construction and professional-ethics practice?

### 2.3 Research purpose and paper structure

Adopting a descriptive-analytical conceptual-speculation paradigm, this study constructs a four-fold theoretical-logical framework for university teachers’ professional ethics cultivation. It clarifies the connotations, interrelations, and functional values of each dimension, providing theoretical references for university teachers’ professional ethics cultivation and ethics-conduct development.

This paper is structured as follows.

Part 1 offers a general overview. Part 2 presents a systematic literature review. Part 3 elaborates transparent research methods. Part 4 visualizes and interprets the four-fold-logic conceptual framework through tables. Part 5 conducts in-depth discussion, comparing the proposed model with existing theories and acknowledging research limitations. Part 6 draws core conclusions and proposes targeted practical countermeasures. Comprehending the intrinsic logic linking professional ethics cultivation to university faculty development helps university administrators respect laws of faculty growth, activate mechanisms through which professional ethics shapes faculty advancement, improve teachers’ overall competencies, and cultivate high-quality teaching teams.

## 3 Method

This paper adopts a qualitative theoretical-research paradigm, relying primarily on philosophical speculation, logical analysis, and textual interpretation to conduct systematic academic inquiry into connections between university teachers’ professional ethics cultivation and their professional development.

### 3.1 Paradigm definition: descriptive-analytical conceptual research

This study falls under descriptive-analytical conceptual research, a form of pure theoretical speculation within educational psychology and teacher-education scholarship. No empirical data are collected. Instead of testing hypotheses through sample statistics, the research aims to deduce and validate intrinsic logical relationships between two core constructs.

This study conceptually organizes and theoretically deduces core connotations, practical orientations, and functional mechanisms of university teachers’ professional ethics cultivation. Through logical construction, relational differentiation, and normative interpretation, it distils and justifies four intrinsic logics whereby professional ethics cultivation shapes faculty development, yielding an integrated analytical perspective covering process factor, professional-attribute internalization, inherent development requirement, and resource endowment.

As theoretical speculation in educational psychology and teacher education, the research excludes empirical-data collection and quantitative analysis. It prioritizes theoretical deduction and logical argumentation to uncover deep-level mechanisms and inherent laws through which professional-ethics cultivation drives faculty development, offering theoretical references and interpretive frameworks for studies on teacher occupational psychology, faculty development, and educational ethics.

### 3.2 Transparent and standardized research procedures

To strengthen methodological rigour and transparency, theoretical construction proceeds through four steps:

1. Systematic literature retrieval and theoretical sorting: Literature on faculty professional ethics cultivation is retrieved and thematically organized around four themes: connotations of professional ethics cultivation, impacts on talent cultivation, practical dilemmas, and optimization pathways.
2. Dimension extraction via logical deduction: Drawing on holistic faculty-growth theories, university teachers’ professional development is decomposed into four progressive layers: individual intensive-development processes, professional-identity formation, endogenous career motivation, and long-term competitive resources. Corresponding to each layer, this paper deduces unique functions of professional ethics cultivation and generates four preliminary logical dimensions.
3. Framework integration and cross-validation: The four preliminary dimensions are cross-referenced against existing scholarship on professional ethics cultivation. Progressive and mutually supportive relationships among the four-fold logics are clarified to form an integrated analytical framework.
4. Argumentation and theoretical interpretation: Each dimension of the four-fold logic is interpreted drawing on domestic and international literature. Dialogue with prior research validates the plausibility of each logical component.

## 4 Results: the complete framework of four underlying logics

### 4.1 Logic 1: professional ethics cultivation as a process factor for the intensive development of higher education teachers

Moral cultivation is an approach for every individual to continuously improve and develop themselves. What constitutes self-improvement and development? From a universal and holistic perspective, moral cultivation includes observing social morality, family virtues, and professional ethics. These three dimensions reflect the inherent requirements of the social, family, and workplace spheres for individuals, respectively. Thus, in the context of an individual’s self-development and improvement, intensive development is centered on the moral dimension. Generally speaking, the universal requirements and value ethics of society, family, and profession constitute the factors for an individual’s intensive development. For the group of higher education teachers—or the profession of higher education teaching—their professional ethics cultivation relates to professional ethics in the workplace. Rooted in the role of higher education teachers, the focus of this cultivation lies in the role dimension. Relying on their professional attributes, professional ethics cultivation has become an indispensable task for higher education teachers in the process of individual development, and it is also the inherent logic for transforming an ordinary individual into a higher education teacher. In short, strengthening the professional ethics cultivation of higher education teachers not only enhances their comprehensive quality but also serves as a nurturing process for individuals to acquire the qualifications of a higher education teacher. Of course, for individuals, professional ethics cultivation is an important manifestation of the improvement of their ideological and moral standards. Consequently, influenced by the differences in professions, the needs of role-playing, external norms, and social expectations, higher education teachers exhibit distinct professional characteristics in professional ethics compared to ordinary individuals. Therefore, teachers’ professional ethics cultivation can be regarded as a process of internalizing the characteristics, principles, and requirements of the teaching profession into individuals, and it is a process factor for the intensive development of higher education teachers. This perspective resonates with Fullan’s account of teacher transformation: comprehensive faculty development cannot rely solely on skill-based training; sustained moral formation must be embedded within ongoing professional growth (Fullan, 2001). Research on teachers’ professional identity further indicates that becoming a qualified university teacher entails continuous reconstruction of role-related self-perception (Beijaard et al., 2004).

### 4.2 Logic 2: professional ethics cultivation as a manifestation of the internalization of higher education teachers’ professional attributes

To build a high-quality teaching team with profound academic accomplishment and good conduct, it is necessary to strengthen the comprehensive education and training of teachers’ morality and ethics. Among these measures, enhancing professional ethics cultivation is not only an external intervention but also a manifestation of the internalization of professional attributes. “Professional ethics literacy refers to the comprehensive quality displayed by a person in their professional position; it is a comprehensive quality that people demonstrate through external behaviors such as individual professional ethics, professional skills, professional conduct, professional style, and professional awareness by internalizing the inherent requirements of professional norms in the process of performing their duties.” (Song, 2019, p. 139). It can be seen that professional ethics cultivation in the training and education of higher education teachers is an internalization of professional attributes. It meets the needs of higher education institutions to strengthen teachers’ morality education, the needs of higher education teachers to improve their professional level, and the needs of students’ growth and development. Specifically, the professional ethics cultivation of higher education teachers refers to “transforming the principles, norms, and requirements of teachers’ professional ethics into teachers’ internal moral qualities to achieve a noble moral realm.” (Song, 2019, p. 141). These needs are all based on the identity of higher education teachers. Without the limitation of this profession, teachers’ moral cultivation would be universal, just like that of other ordinary people. Due to the individual differences among teachers, there are variations in the effectiveness of their professional ethics cultivation. However, the professional requirements and principles reflect the specific domain and role orientation of moral cultivation. That is, professionalism and role orientation are internalized into teachers’ psychological acceptance and recognition through self-cultivation and external education. Therefore, the professional ethics cultivation of higher education teachers is a manifestation of the internalization of their professional attributes. Bandura’s social-cognitive perspective on morality supports this logic: sustained ethics education and self-reflection convert external occupational norms into stable internal cognitive schemas (Bandura, 1991).

### 4.3 Logic 3: professional ethics cultivation as an inherent requirement for teachers’ professional development

The purpose of professional ethics cultivation for higher education teachers is the process of internalizing professional attributes. Of course, in the process of professional development, teachers need to develop and improve in aspects such as teaching skills, teaching techniques, and teaching management. These are external manifestations—variable factors that can be acquired through training. However, the inherent development requirements of teachers cannot be measured by teaching ability, skills, or management alone. Because teachers’ professional ethics cannot be expressed in mathematical terms but in the form of quality and conduct. In other words, in the process of teachers’ professional development, in addition to the visible and measurable external aspects, there are dimensions such as professional ethics that cannot be quantified. This inherent development is an endogenous force in teachers’ professional development, encouraging them to continuously innovate and develop teaching skills and improve teaching ability. Therefore, in teachers’ professional development, the external manifestations are continuously enriched and developed through the promotion of professional ethics. Hence, the professional ethics cultivation of higher education teachers is an inherent requirement for their professional development. From the moral-identity perspective framing moral motivation, teachers with salient moral identities exhibit stronger initiative in instructional reform and research innovation; moral identity supplies irreplaceable endogenous motivation for faculty career advancement (Hardy and Carlo, 2005).

### 4.4 Logic 4: professional ethics cultivation as a resource endowment for teachers’ professional development

The professional development of higher education teachers, from a personal perspective, involves aspects such as teaching ability, teaching skills, and teaching management. This is a narrow understanding of teachers’ professional development. Why? Because teachers’ professional development not only includes the development of qualifications, conditions, and abilities required by the profession but also involves advancements in academic titles, performance evaluations, and administrative positions brought about by the profession. This is a reflection of the connotation and extension of teachers’ professional development, as it is an all-round and comprehensive development. Strengthening the professional ethics cultivation of higher education teachers is a form of psychological construction and development for individual teachers or the collective, and it embodies the intensive development of teachers’ all-round growth. Through professional ethics cultivation, to a certain extent, teachers’ professional potential is nurtured, and their competitiveness in social life-especially in the professional circle—is enhanced through noble professional ethics. Therefore, strengthening the professional ethics cultivation of higher education teachers improves their professional ethics quality, enhances their ability to participate in internal and inter-group competitions, and fosters their potential. Thus, the professional ethics cultivation of higher education teachers is a resource endowment for their professional development. From a human-capital perspective, ethical literacy constitutes intangible human capital for academics that yields long-term competitive advantages in institutional evaluations and academic promotion processes (Becker, 1964).

## 5 Discussion

### 5.1 Comparison with existing theoretical models

Prior scholarship on faculty professional ethics cultivation predominantly adopts one of two isolated perspectives: institutional governance or individual moral-character evaluation; few studies construct integrated multi-dimensional frameworks of driving mechanisms. This paper’s four-fold-logic framework delivers three expansions. First, it builds a progressive logical chain spanning teachers’ full developmental trajectories: starting from ongoing individual growth (Logic 1), moving toward internalization of professional identity (Logic 2), generating endogenous career motivation (Logic 3), and ultimately forming long-term occupational capital (Logic 4). Such progressive linkages receive limited attention in existing literature. Second, the model differentiates four distinct functional values of professional ethics cultivation and overcomes homogenized discussions of moral character prevalent in prior work, respectively defining professional-ethics cultivation as developmental process, carrier of internalization, inherent developmental requirement, and long-term resource endowment. Third, this framework bridges moral-psychology theories and holistic faculty-development theories to produce an interdisciplinary analytical instrument usable in subsequent quantitative and qualitative empirical research.

### 5.2 Synergistic and interdependent relationships among the four-fold logics

The four logics are not independent or fragmented dimensions; rather, they constitute an organically integrated system of mutually supportive, sequentially progressive components. The process factor forms the foundational carrier for all moral growth: only sustained long-term cultivation enables effective internalization of professional attributes. Internalized professional identities further generate endogenous needs for career advancement, for which professional ethics forms the core. Cumulative endogenous moral motivation gradually precipitates into stable intangible occupational resource endowments sustaining teachers’ long-term holistic advancement. Omission of any dimension would undermine the driving effects of professional ethics cultivation on faculty development.

### 5.3 Limitations and future research directions

Rooted in descriptive-analytical conceptual research, this study’s primary limitation lies in the absence of empirical validation. The four-fold-logic framework derives from theoretical deduction. Its explanatory power across different faculty subgroups (early-career versus senior teachers; humanities versus science-and-engineering teachers; teaching-oriented versus research-oriented teachers) requires testing via questionnaires, in-depth interviews, and longitudinal follow-up data.

Two directions are suggested for future research. First, empirical validation: develop measurement scales based on the four dimensions in Table 1 and collect sample data across multiple universities to test structural validity of the model. Second, applied research: drawing on the four-fold-logic framework, design differentiated professional-ethics-cultivation training schemes tailored for distinct faculty subgroups.

**Table 1:** Visualization framework of four underlying logics through which professional ethics cultivation drives university faculty development.

Four underlying logics	Core connotation	Core function for faculty development	Core theoretical foundations
Process Factor for the Intensive Development of Higher Education Teachers	Ethics cultivation constitutes lifelong self-improvement encompassing internalization of social morality, family virtues, and occupational norms; it realizes identity transformation from ordinary individual to qualified university teacher.	Facilitates holistic and intensive faculty growth; elevates overall ideological-moral literacy.	Holistic teacher-growth theory (Fullan, 2001); perspective on teachers’ professional-identity formation (Beijaard et al., 2004)
Manifestation of the Internalization of Higher Education Teachers’ Professional Attributes	Through sustained self-cultivation and institutional training, external public occupational norms for university teachers are internalized into stable psychological acceptance and moral qualities.	Forms occupation-specific professional identities for higher-education practitioners; fulfils moral access criteria for faculty posts.	Social-cognitive theory of morality (Bandura, 1991); perspectives on professional ethics cultivation (Song, 2019)
Inherent Requirement for Teachers’ Professional Development	Professional ethics represents non-quantifiable endogenous spiritual force permeating all observable improvements in teaching and research capacity.	Generates intrinsic motivation for teachers to refine instructional techniques and fulfil educational missions.	Moral-identity perspective on moral motivation (Hardy and Carlo, 2005)
Resource Endowment for Teachers’ Professional Development	Noble professional ethics functions as intangible long-term occupational capital improving comprehensive competitiveness in institutional assessment, academic-rank evaluation, and academic communities.	Accumulates sustainable developmental potential across faculty members’ full career cycles and supports holistic upward advancement.	Human-capital perspective (Becker, 1964)

## 6 Conclusion

In summary, the professional ethics cultivation of higher education teachers exerts a fourfold logic on their development.

*Firstly*, the continuous improvement and development of teachers’ personal self-moral cultivation is a concentrated manifestation of the moral requirements of society, family, and schools, and it constitutes the intensive development of individuals’ all-round growth. Beyond family affairs, the requirements of society and work for individuals are raised to the level of social morality and professional ethics. Therefore, the professional ethics cultivation of teachers supplements and improves their all-round development, and it is an important part of their individual all-round development.

*Secondly*, it is a concentrated manifestation of teachers’ role-playing and positioning, and an internalized expression of the professional requirements, principles, and characteristics of the teaching profession. As a profession, teaching has specific qualification requirements and practical rules, and compared to the private affairs of individuals in the family, it has strong public and professional attributes. The professional ethics cultivation of higher education teachers is based on these professional attributes; it involves providing professional training for practitioners who do not meet teachers’ moral standards to build the moral quality and ability required of qualified teachers.

*Thirdly*, it relates to the endogenous motivation for teachers’ professional development. In addition to the apparent development of teaching skills and techniques, teachers’ professional development also involves the cultivation of internal spirit. Teachers’ sense of responsibility and mission are prominent manifestations of professional ethics. Only through professional ethics cultivation can teachers continuously improve their self-awareness and self-enlightenment, firmly establish the ideological understanding of nurturing people for the people and builders for the modernization drive from the bottom of their hearts, and thereby generate an inherent desire to improve their teaching skills and techniques—which is the inherent requirement for teachers’ professional development.

*Fourthly*, it pertains to the resource endowment for teachers’ professional development. In addition to building a strong endogenous motivation, teachers’ professional development also involves empowering the development of the teacher group. Higher education institutions organize professional ethics training and education for teachers to improve their self-moral cultivation, which continuously enhances their moral quality, accumulates internal motivation for the development of the teacher group, and reserves potential for further development.

Therefore, the professional ethics cultivation of higher education teachers is an important part and manifestation of the teacher group’s self-moral improvement and the realization of individuals’ all-round development. Professional ethics cultivation is a supplementary condition for teachers’ professional access qualifications. Because to become a highly professional teacher from an ordinary individual, in addition to mastering teaching skills, one also needs to possess the psychological quality required of teachers—in other words, professional ethics cultivation is one of the access conditions for teachers. It is a prerequisite and important cornerstone for teachers’ professional development. At the same time, teachers’ professional ethics can to a certain extent stimulate and promote teachers to actively improve and perfect their teaching skills. This is the positive role of professional ethics, and it is also the inherent requirement and endogenous motivation for teachers’ professional development. Teachers’ development should not be limited to a fixed field or level. Strengthening the professional ethics cultivation of higher education teachers accumulates upward momentum for the all-round development of the teacher group (in terms of positions, academic titles, and other aspects) and reserves strategic capabilities for their continuous progress in the future.

### 6.1 Practical implications for teaching and institutional implementation

1. For university administrative departments: Build full-cycle professional-ethics cultivation systems aligned with the four-fold logics. Universities should abandon overreliance on one-off lecture-based training and implement phased cultivation systems matching each logical dimension. For newly recruited teachers, prioritize the first two logics and design progressive long-term moral-practice activities to support intensive growth and professional-identity internalization. For mid-career core teachers, focus on stimulating endogenous developmental motivation and link professional-ethics performance with teaching-innovation incentives. For senior teachers, guide them to accumulate moral-resource endowments and exert exemplary roles in academic evaluation and academic-rank review. Revise faculty assessment indicators to increase weighting for ethical literacy and counterbalance assessment systems overly oriented toward quantitative research outputs.
2. For trainers delivering professional-ethics training: Shift from external doctrinal indoctrination toward facilitating self-directed cultivation. Trainers should reject didactic lecturing and adopt reflective learning, peer mentoring, and case-based seminars to facilitate active internalization of professional ethics. Organize regular professional-ethics reflection workshops to foster teachers’ sustained self-improvement awareness and elicit endogenous career motivation from educational missions, rather than merely distributing normative documents.
3. For individual university teachers: Recognize professional ethics cultivation as core long-term occupational capital. Teachers should discard the misconception that professional-ethics cultivation constitutes an extra burden beyond teaching and research. Treat routine moral self-reflection as an essential component of career growth, actively internalize occupational norms, and translate noble professional ethics into long-term competitive advantages in academic work so that moral literacy, teaching competence, and research capacity advance synergistically.

## Data Availability

The original contributions presented in the study are included in the article/supplementary material, further inquiries can be directed to the corresponding author.
